# Cytotoxicity and Genotoxicity Evaluation of Organochalcogens in Human Leucocytes: A Comparative Study between Ebselen, Diphenyl Diselenide, and Diphenyl Ditelluride

**DOI:** 10.1155/2013/537279

**Published:** 2013-11-21

**Authors:** Diones Caeran Bueno, Daiane Francine Meinerz, Josiane Allebrandt, Emily Pansera Waczuk, Danúbia Bonfanti dos Santos, Douglas Oscar Ceolin Mariano, João Batista Teixeira Rocha

**Affiliations:** Laboratório de Bioquímica Toxicológica, Departamento de Química, Centro de Ciências Naturais e Exatas, Universidade Federal de Santa Maria, Santa Maria 97105-900, RS, Brazil

## Abstract

Organochalcogens, particularly ebselen, have been used in experimental and clinical trials with borderline efficacy. (PhSe)_2_ and (PhTe)_2_ are the simplest of the diaryl dichalcogenides and share with ebselen pharmacological properties. In view of the concerns with the use of mammals in studies and the great number of new organochalcogens with potential pharmacological properties that have been synthesized, it becomes important to develop screening protocols to select compounds that are worth to be tested *in vivo*. This study investigated the possible use of isolated human white cells as a preliminary model to test organochalcogen toxicity. Human leucocytes were exposed to 5–50 **μ**M of ebselen, (PhSe)_2_, or (PhTe)_2_. All compounds were cytotoxic (Trypan's Blue exclusion) at the highest concentration tested, and Ebselen was the most toxic. Ebselen and (PhSe)_2_ were genotoxic (Comet Assay) only at 50 **μ**M, and (PhTe)_2_ at 5–50 **μ**M. Here, the acute cytotoxicity did not correspond with *in vivo* toxicity of the compounds. But the genotoxicity was in the same order of the *in vivo* toxicity to mice. These results indicate that *in vitro* genotoxicity in white blood cells should be considered as an early step in the investigation of potential toxicity of organochalcogens.

## 1. Introduction

 Selenium (Se) is an essential microelement for human and animal nutrition [1]. It is important for selenoprotein synthesis, where it is present as the aminoacid selenocysteine [2]. Several selenoenzymes, such as Glutathione Peroxidase (GPx) and Thioredoxin Reductase (TrxR), are important for the cell defense against oxidative stress [3, 4]. Taking this role of Se in living beings, many therapeutic trials explored the use of inorganic forms of Se as pharmacological agents [5]. However, inorganic forms of Se, such as selenite and selenate, are poorly absorbed and present many toxic effects at high concentrations [6]. Consequently, the interest in organic forms of selenium, that can be less toxic and better absorbed than Se (IV) and Se (VI), has increased.

 Tellurium (Te) is chemically related to Se and can be occasionally found in some proteins in bacteria, yeast, and fungi, but no functional telluroproteins have been found in animal cells [7]. In contrast to Se, Te does not have biological function [8]. However, the literature has demonstrated immunomodulatory, antioxidant, and anticancer properties of various organotellurides [9, 10]. Organotellurium compounds can also mimic Glutathione Peroxidase activity [11], and, consequently, these compounds can be potential antioxidants, effective against some cell damaging agents [12–14].

Ebselen and Diphenyl Diselenide ((PhSe)_2_) are two organoselenium compounds that are recognized as promising pharmacological agents presenting antioxidant, anti-inflammatory, neuroprotective, and other beneficial properties [9]. These compounds can exert their pharmacological effects by mimicking the native Glutathione Peroxidase enzyme (GPx-like activity) or by being a substrate of TrxR. The selenol intermediate formed after their reduction can reduce the levels of reactive oxygen species (ROS) in the cell and prevent oxidative damage to lipids, proteins, and DNA [15–18]. Diphenyl Ditelluride ((PhTe)_2_) is an organotellurium compound that also showed antioxidant and other *in vitro* pharmacological properties [9]. Therefore, the experimental use of organoselenium and -tellurium compounds in different models of human diseases has increased [19–23].

On the other hand, ebselen, (PhSe)_2_, and (PhTe)_2_ can be toxic when administered at high doses. This toxicity is thought to be associated with inhibition of thiol- and/or selenol-containing enzymes, which can increase ROS formation, lipid peroxidation, and DNA damage [24–27]. 

However, the quantity of new organoselenium and -tellurium compounds with pharmacological potential that have been synthesized is increasing rapidly. Consequently, information about the toxicity of new organochalcogens is needed. However, we do not have a simple preliminary test to determine the potential toxicity of a great number of new compounds. This point is critical both in view of the time required to perform assays with vertebrates and the need of ethical adherence to the 3R principal in the use of experimental animals. Here we compare the toxicity of ebselen (which has been used in different clinical trials), (PhSe)_2_ (which is a very simple and pharmacologically active diselenide), and (PhTe)_2_ (a simple and pharmacologically active ditelluride which is also very toxic *in vivo* to rodents) in human white blood cells to determine whether these cells could be used to do a preliminary screening of potentially toxic new organochalcogens.

In short, the aim of this study was to define the cytotoxic concentrations of ebselen, (PhSe)_2_, and (PhTe)_2_ in freshly isolated white human blood cells. Therefore, human leucocytes were exposed to compounds, and their potencial cytotoxic and genotoxic effects were measured using Trypan's Blue Exclusion and Comet Assay Tests.

## 2. Materials and Methods

### 2.1. Chemicals

 Ebselen, (PhSe)_2_, (PhTe)_2_, Trypan's Blue, dextran, and tungstosilicic acid were obtained from Sigma-Aldrich (St. Louis, MO). All the other reagents were obtained from standard chemical suppliers.

### 2.2. Sample Preparation

Leucocytes were isolated from heparinized venous blood obtained from healthy volunteers. The protocol of study was reviewed and approved by the appropriate institutional review board from Guidelines of the Committee of UFSM (0089.0.243.000-07).

2 mL of dextran 5% (dissolved in Phosphate Buffer Saline 1%) was added to 8 mL of blood. The tube was gently mixed and left to stand at room temperature for 45 min. Afterwards, the supernatant was centrifuged (480 ×g, 10 min) and plasma was discarded. The pellet was washed with erythrocyte lysis solution (NH_4_Cl 150 mM; NaHCO_3_ 10 mM; EDTA 1 mM) and centrifuged (480 ×g, 2 min). The supernatant was discarded and the pellet was washed twice with 1 mL erythrocyte lysis solution. After the second centrifugation, the pellet was suspended in 2 mL Hank's buffer solution (KCl 5.4 mM; Na_2_HPO_4_ 0.3 mM; KH_2_PO_4_ 0.4 mM; NaHCO_3_ 4.2 mM; MgCl_2_ 0.5 mM; NaCl 122.6 mM; D-glicose 10 mM, Tris-HCl 10 mM; CaCl_2_ 1.3 mM; pH 7.4). The concentration of leucocytes was adjusted to 2000 cells/*μ*L.

### 2.3. Leucocytes Exposure to Organochalcogens

 Leucocytes were exposed to ebselen, (PhSe)_2_, and (PhTe)_2_ at 5, 10, and 50 *μ*M or an equal volume of DMSO (final concentration of 0.5%) during 3 hours at 37°C. Positive control group was treated with hydrogen peroxide (H_2_O_2_) 2 mM and sodium azide 1 mM.

### 2.4. Trypan's Blue Exclusion Test

 Trypan's Blue exclusion test was performed according to Mischel and Shiingi [28]. After 3 hours of incubation, 50 *μ*L of Trypan's Blue 0.4% was mixed with 50 *μ*L of leucocytes and left to stand at room temperature for 5 minutes. Cell viability was determined microscopically (400x magnification) and expressed as number of viable cells divided by the total number of cells multiplied by 100.

### 2.5. Comet Assay

Comet Assay was performed according to Collins [29] with some modifications. After three hours of incubation, 15 *μ*L of the sample was mixed with 90 *μ*L of low-melting point agarose 0.75% and placed in a slide precoated with agarose 1%. A coverslip was added and the samples were left to solidify at 4°C. The coverslips were removed and the slides were placed on a lysis solution (NaCl 2.5 M; EDTA 100 mM; Tris-HCl 8 mM; Triton X-100 1%; pH 10–10.5) during 24 hours at 4°C. Afterwards, the slides were incubated in an electrophoresis solution (NaOH 300 mM; EDTA 1 mM; pH 13.5) for 20 minutes at 4°C and the electrophoresis was performed (25 V; 300 mA; 7 W) for 20 minutes. All the steps were performed in the dark until this moment. After electrophoresis, the slides were washed in a neutralizing solution (Tris-HCl 400 mM; pH 7.5) three times, washed with distilled water, and left to dry. The slides were rehydrated and fixed (Trichloroacetic acid 15%; ZnSO_4_ 5%; glycerol 5%), washed three with distilled water, and left to dry. Afterwards, the rehydrated slides were stained (Na_2_CO_3_ 5%; NH_4_NO_3_ 0.1%; AgNO_3_ 0.1%; H_4_[W_12_SiO_40_] 0.25%; formaldehyde 0.15%). The slides were immersed in acetic acid 1%, washed, and left to dry.

**Figure 1:**
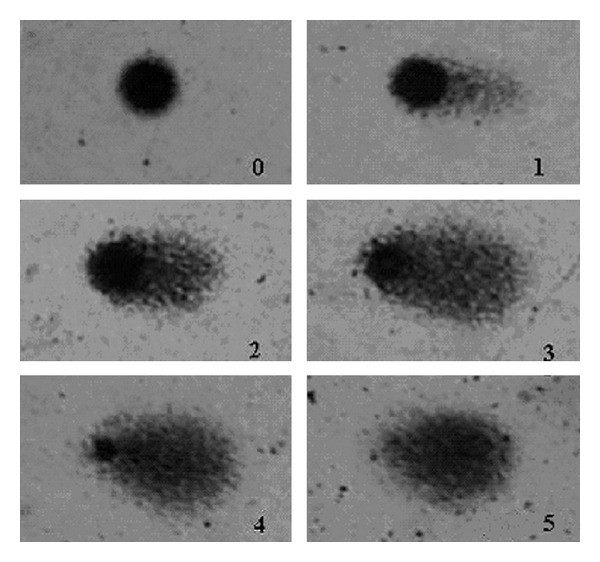
Damage levels considered for analysis in Comet Assay. Level 5 was excluded from our evaluation.

 One hundred cells randomly selected were analyzed in each sample according to tail size and intensity in five classes. The damage score for each cell can range between 0 (no damage) and 4 (maximum damage), according to Figure 1. Damage index (DI) was defined as follows: DI = 1*n*1 + 2*n*2 + 3*n*3 + 4*n*4, where *n*1 represents the number of cells with damage level 1, *n*2 represents the number of cells with damage level 2, *n*3 represents the number of cells with damage level 3, and *n*4 represents the number of cells with damage level 4. At least two different individuals analyzed the slides under blind conditions.

### 2.6. Statistical Analysis

Statistical analyses were performed using analysis of variance (ANOVA) followed by Newman-Keuls multiple test when appropriate. The results are expressed as mean ± SEM for four independent replicates. The difference was considered significant when *P* < 0.05.

## 3. Results and Discussion

Organoselenium compounds, such as ebselen and (PhSe)_2_, are known as pharmacologically active compounds, exhibiting antioxidant, anti-inflammatory, neuroprotective, and antimutagenic properties [9, 20, 22, 30, 31]. At low concentrations, these compounds protect cells against the insults generated by ROS production, depleting H_2_O_2_ via their GPx-mimic activity [32]. In fact, ebselen was used in clinical trials with borderline efficacy [19]. Therefore, the interest in the use of organochalcogens as therapeutic agents has increased in the last years.

Despite their pharmacological properties, organochalcogens can be hepato-, reno-, and neurotoxic to mammals when administered at high doses [33–36]. Accordingly, (PhSe)_2_ administration caused genotoxicity and prooxidant effects in mice [37, 38]. These toxic effects of ebselen, (PhSe)_2_, and (PhTe)_2_ can be secondary to thiol oxidation of critical target proteins, for instance, lactate dehydrogenase [39], Na^+^/K^+^ ATPase [9, 40], and *δ*-aminolevulinic acid dehydratase (*δ*-ALAD) [24, 41, 42]. Recently, we have demonstrated that (PhTe)_2_ can also inhibit important antioxidant selenoenzymes [27].

The data available in the literature about organochalcogens toxicity are scarce, mainly in human cells. So, this study examined comparatively the potential cytotoxic and genotoxic effects of ebselen, (PhSe)_2_, and (PhTe)_2_ in human leucocytes.

**Figure 2:**
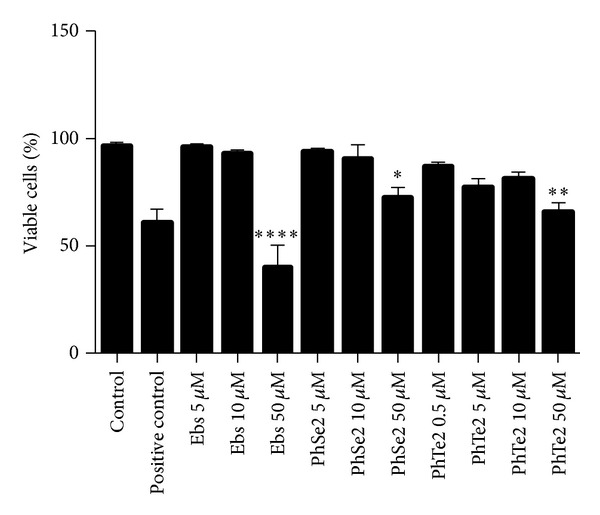
Cellular viability of human leucocytes exposed to organochalcogens for 3 hours. The results are expressed as mean ± SEM from four replicates. One-way ANOVA followed by Newman-Keuls (*P < 0.05, **P < 0.01, and ****P < 0.0001).

DMSO did not modify cell viability. At 50 *μ*M, ebselen, (PhSe)_2_, and (PhTe)_2_ caused a significant decrease in cell viability when compared to the control groups. However, the effect of ebselen (a decrease of about 60%) was higher than that of (PhSe)_2_ (a decrease of about 20%) and that of (PhTe)_2_ (a decrease of about 25% in leucocyte viability, Figure 2). At lower concentrations, ebselen, (PhSe)_2_, and (PhTe)_2_ did not cause significant decrease in cell viability (Figure 2).

**Figure 3:**
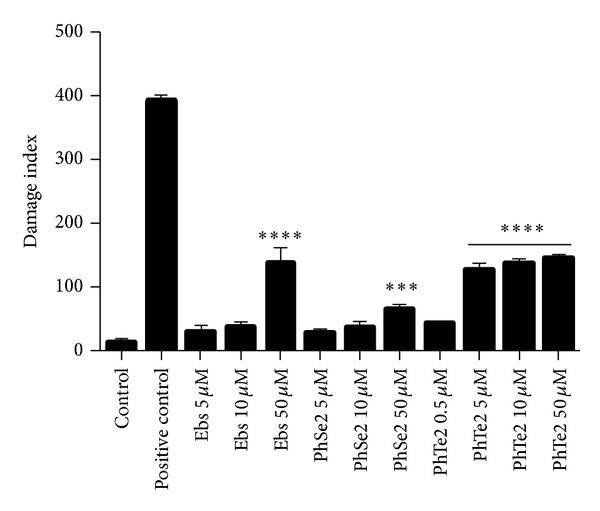
DI of human leucocytes exposed to organochalcogen for 3 hours. Data are expressed as mean ± SEM of four independent experiments done in duplicate. One-way ANOVA followed by Newman-Keuls (***P < 0.001 and ****P < 0.0001).

DMSO did not modify damage index (DI) of DNA in human blood leucocytes. Ebselen and (PhSe)_2_ at 50 *μ*M and (PhTe)_2_ at 5, 10 and 50 *μ*M caused a significant increase in DI when compared to the control group (Figure 3). Statistical analysis indicated that the effect of 50 *μ*M ebselen and (PhTe)_2_ on DI was higher than that caused by (PhSe)_2_ (Figure 3). At 5 and 10 *μ*M, (PhTe)_2_ increased DI, whereas ebselen and (PhSe)_2_ did not cause DNA damage at these concentrations.

Thus, regardless of their structural differences, the toxicity of these compounds can have a common molecular mechanism, that is, oxidation of thiol groups in critical proteins [22, 42, 43]. However, here we observed that ebselen exhibited higher cytotoxicity in human leucocytes than (PhSe)_2_ and (PhTe)_2_. The higher toxicity of ebselen may be related to its capacity to induce thiol oxidation on lactate dehydrogenase [39] and mitochondrial complexes I and II [44] more than (PhSe)_2_ and (PhTe)_2_, which can cause the impairment of cell respiration and, consequently, cell death. Additionally, we observed that ebselen was more genotoxic than (PhSe)_2_, and (PhTe)_2_ was the most genotoxic of the three compounds. A report in the literature shows that (PhTe)_2_ induces cell death via oncosis [45], which is a different type of cell death than that induced by ebselen [9, 46] and (PhSe)_2_ [47]. The different genotoxicity potential may be related to differences in the interaction of these compounds with the reparing DNA machinery, in addition to differences in the reactivity with critical thiol-containing proteins.

## 4. Conclusion

In summary, this study shows that ebselen, (PhSe)_2_, and (PhTe)_2_ can cause cytotoxicity and genotoxicity in human leucocytes, that was expressed, respectively, by a decrease in cell viability in Trypan's Blue exclusion test and an increase of DI in Comet Assay, where the cytotoxic effect of ebselen was more pronounced, while (PhTe)_2_ presented the highest genotoxic effect in freshly isolated human leucocytes. Here, the acute cytotoxicity did not correspond with *in vivo* toxicity of the compounds [9], probably because (PhTe)_2_ induces cell death by a different way than that induced by ebselen and (PhSe)_2_, or otherwise they can have some common steps (for instance, oxidation of thiol proteins, but with different potency and perhaps with some different targets). However, the genotoxicity was in the same order of the *in vivo *toxicity to mice (i.e., (PhTe)_2_ > ebselen > (PhSe)_2_) [9], confirming that the use of Comet Assay in human leucocytes is a good strategy for a preliminary study of genotoxicity. These results indicate that *in vitro* genotoxicity in white blood cells should be considered as an early step in the investigation of potential toxicity of organochalcogens before performing *in vivo* studies with vertebrates. However, more studies are needed to elucidate the toxic effects of ebselen, (PhSe)_2_ and (PhTe)_2_, and their mechanisms of action in different cell types.
